# Endophytic *Paecilomyces formosus* LHL10 Augments *Glycine max* L. Adaptation to Ni-Contamination through Affecting Endogenous Phytohormones and Oxidative Stress

**DOI:** 10.3389/fpls.2017.00870

**Published:** 2017-05-29

**Authors:** Saqib Bilal, Abdul L. Khan, Raheem Shahzad, Sajjad Asaf, Sang-Mo Kang, In-Jung Lee

**Affiliations:** ^1^School of Applied Biosciences, Kyungpook National UniversityDaegu, South Korea; ^2^UoN Chair of Oman’s Medicinal Plants and Marine Natural Products, University of NizwaNizwa, Oman

**Keywords:** soybean, nickel stress, endophytic fungi, phytohormones, antioxidant enzymes, fatty acids

## Abstract

This study investigated the Ni-removal efficiency of phytohormone-producing endophytic fungi *Penicillium janthinellum*, *Paecilomyces formosus*, *Exophiala* sp., and *Preussia* sp. Among four different endophytes, *P. formosus* LHL10 was able to tolerate up to 1 mM Ni in contaminated media as compared to copper and cadmium. *P. formosus* LHL10 was further assessed for its potential to enhance the phytoremediation of *Glycine max* (soybean) in response to dose-dependent increases in soil Ni (0.5, 1.0, and 5.0 mM). Inoculation with *P. formosus* LHL10 significantly increased plant biomass and growth attributes as compared to non-inoculated control plants with or without Ni contamination. LHL10 enhanced the translocation of Ni from the root to the shoot as compared to the control. In addition, *P. formosus* LHL10 modulated the physio-chemical apparatus of soybean plants during Ni-contamination by reducing lipid peroxidation and the accumulation of linolenic acid, glutathione, peroxidase, polyphenol oxidase, catalase, and superoxide dismutase. Stress-responsive phytohormones such as abscisic acid and jasmonic acid were significantly down-regulated in fungal-inoculated soybean plants under Ni stress. LHL10 Ni-remediation potential can be attributed to its phytohormonal synthesis related genetic makeup. RT-PCR analysis showed the expression of *indole-3-acetamide hydrolase*, *aldehyde dehydrogenase* for indole-acetic acid and *geranylgeranyl-diphosphate synthase*, *ent-kaurene oxidase* (*P450-4*), *C13-oxidase* (*P450-3*) for gibberellins synthesis. In conclusion, the inoculation of *P. formosus* can significantly improve plant growth in Ni-polluted soils, and assist in improving the phytoremediation abilities of economically important crops.

## Introduction

Rapid industrialization has contributed to the increased heavy metal pollution in the environment (Qin et al., 2015; Wu et al., 2015). Effluents from industry mix with rivers and drains, which enter the food chain through crops grown in contaminated water. Consequently, by consuming contaminated foods, heavy metals accumulate in the human body and lead to severe health problems, including cardiac disorders, digestive disorders, and kidney, stomach, liver, and lung cancer (Qin et al., 2015). Heavy metal pollutants in the soil are toxic and non-biodegradable and are thus extremely persistent and stable in the environment (Wu et al., 2015). Therefore, their removal or conversion to less toxic forms in the water-soil is deemed crucial in order to provide land that is safe and free from heavy metal pollution for agriculture purposes (Amari et al., 2014).

Serious concerns have been raised in developing countries regarding environmental pollution caused by heavy metals. Among heavy metals, trace amounts of nickel (Ni) are required by plants for the regulation of different metabolic pathways (Yusuf et al., 2011; Amari et al., 2014). However, exposure to excess amounts of Ni is toxic to plants, and induces lethal alterations in plant metabolism resulting in the inhibition of plant growth, wilting, necrosis, and chlorosis (Amari et al., 2014). Consequently, plant growth, yield, and quality are drastically affected, and the consumption of Ni-contaminated food can lead to serious health problems (Amari et al., 2014; Clemens and Ma, 2016). Therefore, bio-remediation is an efficient and inexpensive technique used to detoxify or remove Ni contamination from the soil and to provide land that is free from Ni toxicity for crop cultivation. This represents an alternative way of providing safe food for humans, thus preventing the development of lethal disorders induced by Ni toxicity (Kamran et al., 2015; Stella et al., 2017). Hence, the development of economical and reliable strategies is required for the removal or detoxification of heavy metals in order to avoid metal pollution of the environment and to provide safe food for human consumption.

In this regard, interactions between microbes and plants in soil may lead to the development of symbiosis, owing to the potential role of microorganisms in eliminating the toxic effects of metal-contaminated soil and their possible role in augmenting plant growth in metal-contaminated soil by providing nutrients and metabolites to the plant (Khan and Lee, 2013). Endophytes can play a pivotal role in the bioremediation of soils rich in heavy metals, and are considered a cost-effective and favorable replacement of conventional physical and chemical based treatments (Deng et al., 2011; Khan and Lee, 2013). Various studies have recognized that endophytic microbes can significantly enhance the host plant’s potential to grow in soil polluted with heavy metal (Deng et al., 2016). The ability of plants to survive in heavy metal-contaminated soil is attributable to the positive role played by endophytic fungi in the detoxification and degradation/removal of heavy metals from soil, as well as the promotion of enhanced host plant growth due to the production of growth regulators (Deng et al., 2016). Different endophytic microbes such as *Rhizodermea veluwensis* and *Phialocephala fortinii* fungi, and *Cryptococcus* sp., *Enterobacter* sp., and *Bacillus thuringiensis* bacteria have been identified for enhancing bioremediation in metal-polluted soil (Deng et al., 2016; Ma et al., 2016). Such symbiotic interactions between plants and phytohormone-producing endophytes have drawn significant attention owing to their biotechnological potential for mitigating and degrading heavy metals from polluted media and promoting growth and yield in metal contaminated soils.

Among the endophytic microbes, fungi are considered superior to bacteria owing to their ubiquitous, multifaceted, morphological diverse nature and higher capacity of tolerance to environmental stresses (Kaushik and Malik, 2010, 2011; Mishra and Malik, 2014). Moreover, endophytic fungi tend to produce plant growth regulators such as auxins and gibberellins (GAs), and protect plants from both biotic and abiotic stresses (Khan et al., 2015a; Deng and Cao, 2017). Similarly, they have been known to produce exopolysaccharides, proteins, extracellular enzymes, organic acids, and other metabolites that aid the removal of soil pollutants by enhancing the phytoremediation capacity of host plants (Khan et al., 2011a; Redman et al., 2011). Endophytic fungi possess appropriate metal chelation or sequestration abilities, which boost their level of tolerance to heavy metals as well as their sophisticated multicellular biomass making them suitable for use in bioremediation (Aly et al., 2011). In addition, endophytic fungi have coined for the production of phytohormones such as GA, auxin (indole acetic acid – IAA) and abscisic acid etc, in recent decade or so, owing to their potential benefits in plant stress physiology (Khan et al., 2015a). Despite these traits, the role of plant growth-promoting endophytic fungi in the context of bioremediation has not been extensively explored (Bourdel et al., 2016). Therefore, understanding the association of bioaccumulating metal from contaminated soil, phytohormone-producing endophytic fungi with plants, will not only excel metal-tolerance capabilities/phytoremediation but will also promote plant growth and yield.

*Glycine max* (Soybean) growth from germination through to yield is usually affected by the lethal effects of various metal/metalloids including Ni toxicity in the soil, which consequently inhibits normal plant growth by adversely targeting adsorption, translocation, and synthesis processes (Pérez-Chaca et al., 2014; Miransari, 2015; Abdel Latef et al., 2016). Soybean is one of the best agricultural crops, especially in East-Asian countries, owing to its outstanding medicinal and nutritional values (Miransari, 2015). Therefore, we aimed to screen various phytohormone-producing endophytic fungi in order to assess their potential for bioaccumulating metals in the contaminated medium, as well as their ability to alleviate heavy metal toxicity in host plants. The following fungal endophytes were selected: *Penicillium janthinellum* LK5, *Paecilomyces formosus* LHL10, *Exophiala* sp. LHL08, and *Preussia* sp. BSL10, and screened for their metal accumulation potential against different heavy metals (**Table 1**). Based on the production of GA and other phytohormones/secondary metabolites associated with plant defense, such as IAA, abscisic acid, and proline, the most bioactive fungal strain in terms of metal accumulation was selected for interaction with host soybean plants in metal-polluted soil in order to elucidate its metal-removing capacity and its effects on soybean plant growth and physiology.

**Table 1:** List of endophytic fungi with their traits used for initial screening of assessing their metal metals tolerance.

Endophytic fungi	Isolated host	Bioactive metabolites	Accession no.	Effect on plant growth under abiotic stress	Reference
*Penicillium janthinellum* LK5	Tomato	Gibberellins	JX111908	Growth promoting	Khan et al., 2013, 2014
*Paecilomyces formosus* LHL10	Cucumber	Auxin and gibberellins	HQ444388	Growth promoting	Khan et al., 2012a
*Exophiala* sp. LHL08	Cucumber	Gibberellins	HM017066	Growth promoting	Khan et al., 2011a
*Preussia* sp. BSL10	Frankincense	Auxin and extracellular enzymes (gibberellins not published)	KR231682	Growth promoting	Khan et al., 2016


## Materials and Methods

### Endophytes Growth and Their Tolerance against Heavy Metal

*Penicillium janthinellum*, *P. formosus*, *Exophiala* sp., and *Preussia* sp. were isolated from the roots of tomato, cucumber, and frankincense plant leaf, respectively (**Table 1**). The endophytes were previously identified by extracting genomic DNA, PCR amplification of the internal transcribed spacer (ITS) region, and constructing a phylogenetic tree (Khan et al., 2011b, 2012a, 2014; 2016). The universal primers used for the identification were ITS-1 (5′-TCC GTA GGT GAA CCT GCG G-3′) and ITS-4 (5′-TCC TCC GCT TAT TGA TAT GC-3′) and LR0R (F) (5′-ACC CGC TGA ACT TA AGC-3′) and TW13(R) (5′-GGT CCG TGT TTC AAG ACG-3′).

The aforementioned strains were grown on potato dextrose agar (PDA) plates supplemented with 1 mM copper (CuSO_4_), cadmium (CdSO_4_), and nickel (NiSO_4_), respectively. The PDA plates were then incubated at 28°C in for 7 days in darkness, after which the growth rate of endophytes was measured as described by Khan et al. (2017a). The most active metal resistant fungal strain was grown in broth and subjected for ICP-MS analysis to determine metal uptake potential. The bioactive strain was selected based on primary screening and grown in Czapek broth for 10 days at 30°C in a shaking incubator with 120 rpm for further experiments.

### RNA Isolation and Reverse Transcription-PCR

In order to validate IAA and GA producing capability, the expression of IAA and GAs related genes of most bioactive fungal strain was carried out. For this purpose, the extraction of total RNA was performed from endophytic fungal according to the method described by Marcos et al. (2016). Briefly, fungal mycelia (0.1–0.2 g) were disrupted in 1 ml of TRI reagent (Sigma) with 1.5 g of zirconium beads by using a cell homogenizer. Cell debris was removed by centrifugation and supernatants were obtained with chloroform. RNA was precipitated with isopropanol and treated with DNase I (USB) prior to use in RT-PCR experiments. To synthesize cDNA, 1.0 μg of total RNA was then used by using a DiaStar^TM^ RT Kit (SolGent, South Korea) according to manufacturer’s standard protocol. The expression of IAA and GAs related genes were compared with relative expression of actin as internal control. The detail list of genes and their primers are given in Supplementary Table S1.

### Endophyte and Host–Plant Interactions under Heavy Metal Stress

Seeds of soybean (Taekwang Cultivar) were surface-sterilized by washing with 70% ethanol for 30 s followed by 2.5% sodium hypochlorite for 30 min and several rinsing steps with autoclaved double distilled water. Then, seeds were incubated at 28°C for 24 h to obtain equal germination. To investigate the plant–microbe association under metal toxicity, the germinated seeds were sown in autoclaved pots containing 1 kg of horticulture soil composed of cocopeat (68%), perlite (11%), zeolite (8%), as well as micronutrients available as NH^4+^ ∼0.09 mg g^-1^; P_2_O_5_ ∼0.35 mg g^-1^; NO_3_^-^ ∼0.205 mg g^-1^; and K_2_O ∼0.1 mg g^-1^.

The metal-resistant bioactive strain grown in Czapek broth was applied to the plants (25 ml per pot, with 3–4 g of fungal mycelia) sown in pots as described by Khan and Lee (2013) to initiate the symbiotic association between fungus and plant. The non-inoculated plants were treated with the same amount of fungal-free Czapek broth in order to stabilize the effect of additional nutrients on plants. Thus, the plants were grown inside a growth chamber (day/night cycle: 14 h; 28°C/10 h; 24°C; relative humidity 60–70%; light intensity 1000 Em-2-s Natrium lamps) for 15 days. Then, different concentrations of Ni (0.5, 1, and 5 mM) were applied to the soybean plants. After every 24 h, 80 ml Ni solution of each respective concentration was applied to the plants for 2 weeks. To avoid the leaching of metal, each plant was irrigated before Ni treatment. The experimental design involved the following treatments: (i) control fungi free media treated plants without metal stress; (ii) endophytic fungal-treated plants without metal stress, (iii) metal-treated plants (0.5, 1, or 5 mM); (iv) fungal-inoculated plants with in addition to Ni stress (as above). The experiment was replicated three times, with 15 replicates per treatment.

### Growth Attributes of Plants under Nickel Stress

After 15 days of stress, the chlorophyll content of plants subjected to each treatment was measured by employing a chlorophyll meter (SPAD-502 Minolta, Japan). Immediately after harvesting, plant shoot and root lengths, as well as fresh weight, were measured and samples were stored at -80°C for different biochemical analyses. The dry weights were measured after drying in an oven at 65°C for 72 h.

### Analysis of Ni in Fungal Mycelium and Plant Roots and Shoots through ICP-MS

To analyze the root and shoot nickel contents well as in fungal mycelium, the freeze-dried samples were processed into a powder and subjected to the quantification of Ni by inductively coupled plasma atomic emission spectroscopy (ICP) (Optima 7900DV, PerkinElmer, United States). The translocation efficiency of Ni from the root-to-shoot was measured by calculating the translocation factor (TF) using the following formula: TF = total concentration of Ni in shoots mg plant^-1^)/total concentration of Ni in root mg plant^-1^. Similarly, the nickel tolerance index (TI) of plants was assessed as determined by Khan et al. (2017b) using the following formula: TI % = (root length in Ni treatment/root length in control) × 100.

### Lipid Peroxidation and Fatty Acid Quantification of Plants under Ni Stress

The level of lipid peroxidation was analyzed as reported by Khan et al. (2013). Briefly, plant shoots ground with liquid nitrogen were extracted with 10 mM phosphate buffer at pH 7. The reaction mixture was prepared by adding 0.2 ml 8.1% sodium dodecyl sulfate, 1.5 ml 20% acetic acid (pH 3.5), and 1.5 ml 0.81% thiobarbituric acid aqueous solution to the supernatant. The reaction mixture was heated in boiling water for 60 min, immediately after cooling to room temperature, 5 ml solution butanol: pyridine (15:1 v/v) was added. The upper layer of organic acid was removed, and the optical density of the resulting pink color was measured at 532 nm using a spectrophotometer. The level of lipid peroxidation was expressed as the amount of malondialdehyde (MDA) formed per gram tissue weight. The experiment was performed in triplicate.

The fatty acid profile (relative percentage of total fatty acid) of randomly selected plants from each treatment was determined by following the protocol reported by Khan et al. (2012b). Briefly, each plant sample (1 g) was treated with 10 ml of hexane and kept in a shaking incubator (150 rpm) at 50°C for 2 days. Centrifugation (1200 × *g* at 25°C) was outperformed to separate the supernatant, which was then transferred into new tubes. Hexane was evaporated from each sample by passing air through an evaporating unit. The extracted material from each sample was placed in a screw-capped vial, and 5 ml of methylation solution (H_2_SO_4_:methanol:toluene = 1:2:10 ml) was added. Then, the sealed vials were placed in a water bath (100°C) for 60 min for heating, followed by cooling at room temperature. Then, water (5 ml) was added and the samples were shaken. Two layers formed, and were separated by taking the upper layer and subjecting it to dryness using anhydrous sodium sulfate for 5 min. The sample (1 μl) was directly injected to the GC using an automatic sampler (Agilent 7683B). GC–MS analysis was carried out on an Agilent Model 7890A series (Agilent, Dover, DE, United States) equipped with an Agilent 5975C MS detector, an Agilent 7683 autosampler, and a MS ChemStation Agilent v. A.03.00. GC–MS was equipped with a DB-5MS capillary column (30 m × 0.25 mM i.d. × 0.25 μm film thickness; J&W Scientific-Agilent, Folsom, CA, United States) while helium was used as a carrier gas with a flow rate of 0.6 ml/min and a split mode [1:50]. The injector and detector temperature were 120 and 200°C, respectively. The column temperature was programmed from 50 to 200°C at 10°C/min and then finally held at 200°C for 5 min. The mass conditions were: ionization voltage, 70 eV; scan rate, 1.6 scan/s; mass range, 30–450; ion source temperature, 180°C. The components were identified based on the comparison of their relative retention time and mass spectra with those of standards, Wiley7N, NIST library data of the GC–MS system, and published data. The samples were assessed for palmitic acid, stearic acid, oleic acid, and linolenic acid.

### Analysis of Antioxidant and Related Enzymes in Inoculated and Non-inoculated Plants

Reduced glutathione (GSH) content was measured by Ellman (1959) protocol as described by Khan et al. (2015b) with slight modifications. Briefly, 100 mg fresh leaf tissue was ground in 3 ml of 5% (v/v) trichloroacetic acid using a chilled mortar and pestle. The homogenate was subjected to centrifugation at 12,000 rpm for 15 min at 4°C. The obtained supernatants were used to analyze the GSH contents by taking 0.1 ml of the sample supernatant and mixing with 3.0 ml 150 mM monosodium phosphate buffer (pH 7.4) and 0.5 ml of Ellman’s reagent. For each reaction, the mixture was incubated at 30°C for 5 min. The absorbance was measured at 412 nm and the GSH content was calculated using a standard curve.

The activity of antioxidant enzymes such as peroxidase (POD) and polyphenol oxidase (PPO) was analyzed by the method reported by Khan et al. (2017a) with slight modifications. Briefly, leaf samples (400 mg) were ground using a chilled mortar and pestle. Then, the samples were homogenized with 0.1 M potassium phosphate buffer (pH 6.8) and centrifuged at 4°C for 15 min at 5000 rpm in a refrigerated centrifuge. The reaction mixture for the POD assay contained 0.1 M potassium phosphate buffer (pH 6.8), 50 μl pyrogallol (50 μM), 50 μl H_2_O_2_ (50 μM), and 100 μl of the sample crude extract. The reaction mixture was incubated for 5 min at 25°C, followed by the addition of 5% H_2_SO_4_ (v/v) in order to stop the enzymatic reaction. The level of purpurogallin formed was determined by the absorbance at 420 nm. For the PPO activity assay, the same reaction mixture containing the same components as that used for POD excluding H_2_O_2_ was used and the resulting assay was measured at 420 nm. One unit of POD or PPO was measured as a 0.1-unit increase in absorbance. Superoxide dismutase (SOD) activity was measured according to method described by Sirhindi et al. (2016), following the photo reduction of nitroblue tetrazolium (NBT). The absorbance was measured by spectrophotometer at 540 nm. SOD unit is the quantity of enzyme that hampers 50% photo reduction of NBT and is expressed as U/mg protein. Catalase (CAT) activity was assessed by the method reported by of Sirhindi et al. (2016). The absorbance was measured by spectrophotometer at 240 nm and the activity was expressed as U/mg protein

### Endogenous Abscisic Acid (ABA) and Jasmonic Acid (JA) Quantification

The endogenous ABA contents were extracted following the method described by Qi et al. (1998). ABA was extracted from the plant roots and shoots by an extraction solution containing 95% isopropanol, 5% glacial acetic acid, and 20 ng of [(±)-3,5,5,7,7,7-d6]-ABA. The extract was filtered and then concentrated via a rotary evaporator. The residue was dissolved in 4 ml 1 N sodium hydroxide solution, and then rinsed three times with 3 ml of methylene chloride in order to remove lipophilic materials. After reducing the pH of the aqueous phase to approximately 3.5 by adding 6 N HCl, the sample was partitioned through solvent-solvent extraction with ethyl acetate (EtOAc) three times. EtOAc extracts were then combined and evaporated. The nearly dry residue was dissolved in phosphate buffer solution (pH 8.0), which was run through a polyvinylpolypyrrolidone (PVPP) column. The eluted phosphate buffer solution was adjusted to pH 3.5 with 6 N HCl and again partitioned three times into EtOAc. All three EtOAc extracts were combined again and evaporated through a rotary evaporator. The residue was dissolved in dichloromethane (CH2Cl2), and passed through a silica cartridge (Sep-Pak; Water Associates, Milford, MA, United States), which was pre-washed with 10 ml of diethyl ether: methanol (3:2, v/v) and 10 ml of dichloromethane. ABA was recovered from the cartridge by elution with 10 ml of diethyl ether (CH3-CH2)2O: methanol (MeOH) (3:2, v/v). The resulting extract was dried with N_2_ gas and subsequently methylated by adding diazomethane for GC–MS analysis using selected ion monitoring (SIM) 6890N network GC system, and the 5973 network mass-selective detector; Agilent Technologies, Palo Alto, CA, United States). The monitor responses to ions of *m*/*z* of 190 and 162 for Me-ABA, and 194 and 166 for Me-[^2^H_6_]-ABA, were obtained using Lab-Base (ThermoQuest, Manchester, United Kingdom) data system software.

For the quantification of endogenous JA, the protocol reported by McCloud and Baldwin (1997) was followed. The freeze-dried stem and root tissues were separately ground to powder with a chilled mortar and pestle, and 0.1 g of the ground powder was suspended in a mixture of acetone and 50 mM citric acid (70:30, v/v). Internal standard [9,10-^2^H_2_]-9,10-dihydro-JA (20 ng) was also added to the suspension. The extracts were left overnight at a low temperature to permit the highly volatile organic solvent to evaporate, retaining the less volatile fatty acids. The resulting aqueous solution was filtered, and then extracted three times with 10 mL diethyl ether. The combined extracts were then loaded onto a solid-phase extraction cartridge (500 mg of sorbent, aminopropyl), and the cartridges were washed using 7.0 mL of trichloromethane and 2-propanol (2:1, v/v). The exogenous JA and relevant standard were eluted with 10 mL of diethyl ether and acetic acid (98:2, v/v). After evaporation of the solvents, the residue was esterified with excess diazomethane, the volume was adjusted to 50 μL with dichloromethane, and the extracts were analyzed by GC–MS (6890N network GC system and the 5973 network mass selective detector; Agilent Technologies, Palo Alto, CA, United States) in the selected ion mode. The ion fragment was monitored at *m*/*z* = 83 amu corresponding to the base peaks of JA and [9,10-^2^H_2_]-9,10-dihydro-JA; the amount of endogenous JA was estimated from the peak areas compared with the respective standards. The whole experiment was performed three times.

### Statistical Analysis

Experiments were independently performed in triplicate and the values obtained are presented as the means ± standard deviation (SD). Data obtained showing the effect of Ni toxicity on the growth attributes of soybean were subjected to *t*-test using online GraphPad Prism software to determine the significant difference among treatment means at *P* < 0.05. The biochemical analyses were analyzed with two-way analysis of variance (ANOVA) using GraphPad Prism software (version 6.01, San Diego, CA, United States).

## Results

### *In Vitro* Screening of Endophytic Fungi against Heavy Metals Uptake

**FIGURE 1:**
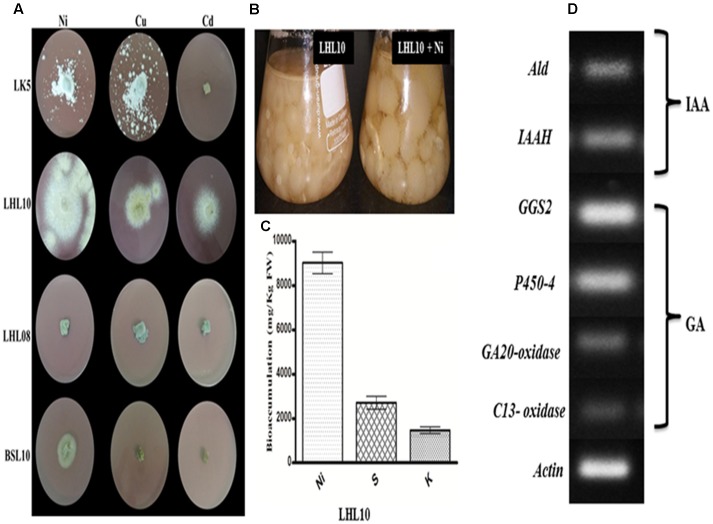
**Growth dynamics of selected fungal endophytes in 1.0 mM Ni, Cu, Cd amended mediums.** **(A)** Each fungus was screened against metals toxicity and their ability to survive. The diameter of growing mycelia was measured after 7 days of incubation at 25°C. **(B)** On the base of significant growth in Ni stress, LHL10 was grown in PDB medium for metal and nutrient uptake analysis. **(C)** Metal uptake analysis via ICP-MS. **(D)** Genes expression related to gibberellins (GAs) and indole acetic acid (IAA) biosynthesis of *P. formosus*. LHL10. *Indole-3-acetamide hydrolase* (IAAH), *aldehyde dehydrogenase* (*Ald*) are IAA related genes, while GAs related genes are *geranylgeranyl-diphosphate synthase* (GGS2), *ent-kaurene oxidase (P450-4)*, *GA 20-oxidase* and *C13-oxidase (P450-3)*. The **(A)** is representative of five replications whereas **(B)** and **(C)** are repeated three times.

The results revealed that among four endophytic fungal strains, *Exophiala* sp. and *Preussia* sp. are highly sensitive to Ni, Cu, and Cd toxicity, which significantly inhibited their growth, with the exception of *Preussia* sp. showed mild growth in Ni-amended PDA medium with 120 mm^2^ growth area (**Figure 1**) Contrarily, *P. formosus* and *P. janthinellum* had substantial tolerance to Ni toxicity followed by Cu with growth area of 424 ± 0.53, 135 ± 0.62, 230 ± 0.54, and 190 ± 0.32 mm^2^, respectively. In terms of Cd toxicity, only *P. formosus* exhibited trivial resistance with a growth area of 120 mm^2^. Thus, following initial investigations of tolerance, and owing to its low-to-high growth trend under Cd, Cu, and Ni toxicity, *P. formosus* was further analyzed in plant–microbe associations under Ni stress. Upon significant growth of *P. formosus* LHL10 in Ni-amended PDA medium, we grow it in PDB supplemented with Ni (**Figure 1B**) in order to evaluate its metal accumulating potential through ICP-MS quantification. Results revealed that fungal grown in PDB medium had significantly accumulated heavy metal in mycelia (**Figure 1C**). Along with heavy metal accumulation, fungal mycelium exhibited significant potential of accumulating sulfur (S) and potassium (K) contents. Since, such improved growth of fungi during stress conditions have been attributed to their potential to produce bioactive secondary metabolites and previous studies have shown this (Khan et al., 2015b), therefore, to further validate the phytohormones producing role of *P. formosus* LHL10, we have carried out RT-PCR analysis to know the existence of IAA and GA related biosynthesis pathways.

### Expression of IAA and GA Biosynthesis Related Genes of *P. formosus* LHL10

The results related to the expression of IAA and GA biosynthesis genes in *P. formosus* LHL10 is shown in (**Figure 1D**). The IAA producing ability of *P. formosus* was previously quantified by HPLC (Khan et al., 2012a), which was further validated in the current study by the real time RT-PCR analysis. The results confirmed that the LHL10 showed the ability to produce both IAA and GA. In case of IAA, we observed the expression of its biosynthesis related genes, i.e., *aldehyde dehydrogenase (ALD) and indole-3-acetamide hydrolase (IAAH)*. Similarly, in case of GA biosynthesis *geranylgeranyl-diphosphate synthase (GGS2)*, *ent-kaurene oxidase (P450-4)*, *GA20-oxidase*, and *C13-oxidase* expressions were found and the results exhibited induced transcript accumulation in RT-PCR, recommending the existence of GA genes cluster in *P. formosus*. However, the expression of *(GGS2)* and *(P450-4)* was comparatively higher than *GA20-oxidase* and *C13-oxidase.*

### Association of *P. formosus* LHL10 under Ni Stress Enhance Plant Growth and Chlorophyll Content

**FIGURE 2:**
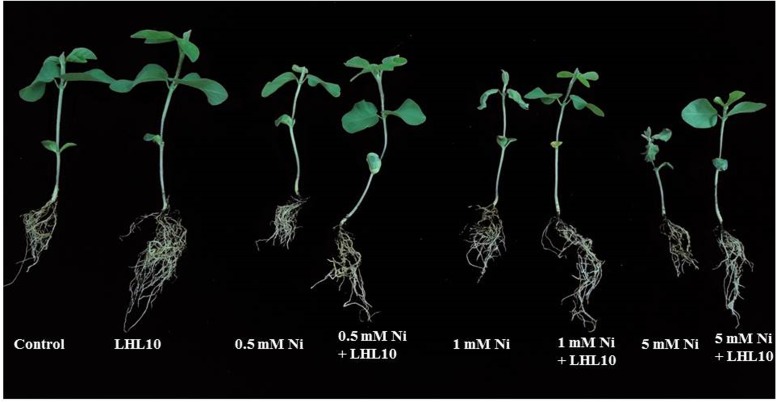
**Effect of endophytic fungus on plant growth under Ni stress.** Each plant represents 15 replicates of eight treatments.

The results of the current study clearly demonstrated a significant growth effect (*p* < 0.05) for soybean plants inoculated with endophytic fungi under different levels of Ni stress. The association of soybean plants with *P. formosus* significantly enhanced growth by stimulating an increase in the length and fresh/dry weight of shoot and root well as chlorophyll content in comparison to non-inoculated plants (**Figure 2** and **Table 2**). Soybean plants subjected to Ni stress exhibited considerable retardations in shoot, root length, and fresh and dry weight, which adversely affected endophytic fungal-free plants. Furthermore, the interaction between fungus and plant mitigated Ni toxicity and promoted enhanced shoot and root lengths, and fresh and dry weights in plants grown with low, medium, and high concentrations of Ni compared with those of non-endophytic-infected plants. With heavy metal application, shoot and root lengths, and fresh and dry weights were significantly (*p* < 0.05) higher (ranging from 11 to 47%) in endophytic fungal-treated plants compared to control plants under varying concentrations of Ni stress (**Table 2**). The chlorophyll contents in both endophytic fungal-inoculated and non-inoculated plants under different concentrations of soil Ni were screened. *P. formosus* plants exhibited an approximate 10% increase in chlorophyll levels compared with the non-inoculated plants in the absence of Ni toxicity. A marked reduction in the chlorophyll content was detected as the level of Ni toxicity in the soil increased. Non-inoculated plants displayed reductions of 13, 26, and 46% under low, medium, and high levels of Ni toxicity, respectively, in the soil compared with inoculated plants (**Table 2**).

**Table 2:** Growth promoting effect of *Paecilomyces formosus* LHL10 in soybean under various nickel stress.

Treatment	SL (cm)	RL (cm)	SFW (g)	SDW (g)	RFW (g)	RDW (g)	CC (SPAD)	TF	TI%
Control	24.46 ± 0.38^b^	13.27 ± 0.58^b^	14.71 ± 0.61^b^	5.47 ± 0.62^b^	6.19 ± 0.52^b^	2.16 ± 0.22^b^	31.74 ± 1.52^b^	–	–
Fungus	26.59 ± 0.86^a^	16.63 ± 0.46^a^	16.74 ± 0.73^a^	6.22 ± 0.49^a^	7.25 ± 0.61^a^	2.87 ± 0.35^a^	34.91 ± 1.12^a^	–	108.67
0.5 mM Ni	20.44 ± 0.25^b^	12.77 ± 0.63^b^	12.99 ± 0.65^b^	3.97 ± 0.38^b^	5.25 ± 0.68^b^	1.71 ± 0.27^b^	24.37 ± 1.14^b^	0.58	83.55
Fungus + 0.5 mM Ni	23.13 ± 0.53^a^	14.81 ± 0.61^a^	17.18 ± 0.78^a^	4.82 ± 0.51^a^	8.92 ± 0.45^a^	2.69 ± 0.41^a^	29.77 ± 1.35^a^	0.62	94.56
1 mM Ni	19.43 ± 0.84^b^	9.15 ± 0.89^b^	11.42 ± 0.47^b^	3.55 ± 0.32^b^	4.75 ± 0.56^b^	1.57 ± 0.43^b^	20.54 ± 1.39^b^	0.61	79.41
Fungus + 1 mM Ni	22.01 ± 0.81^a^	13.17 ± 0.63^a^	15.46 ± 0.01^a^	4.50 ± 0.62^a^	8.74 ± 0.46^a^	2.54 ± 0.49^a^	27.12 ± 0.76^a^	0.52	89.98
5 mM Ni	16.4 ± 0.89^b^	6.21 ± 0.81^b^	10.51 ± 0.76^b^	2.98 ± 0.43^b^	3.88 ± 0.52^b^	1.15 ± 0.37^b^	12.52 ± 0.79^b^	0.56	67.02
Fungus + 5 mM Ni	19.04 ± 0.74^a^	11.85 ± 1.32^a^	14.25 ± 0.98^a^	3.94 ± 0.49^a^	6.98 ± 0.60^a^	2.07 ± 0.15^a^	22.56 ± 1.14^a^	0.53	77.84


*SL, shoot length; RL, root length; SFW, seedlings fresh weight; SDW, shoot dry weight; CC, chlorophyll content; TF, translocation factor; TI, percentage of tolerance index. Each value represents mean ± SD of 15 replicates from three independent experiments. Values in the columns followed by different letters are significantly different at *p* ≤ 0.05 based on *t*-test.*

### Influence of *P. formosus* Inoculation on Metal Uptake and Distribution in Soybean Plant

**FIGURE 3:**
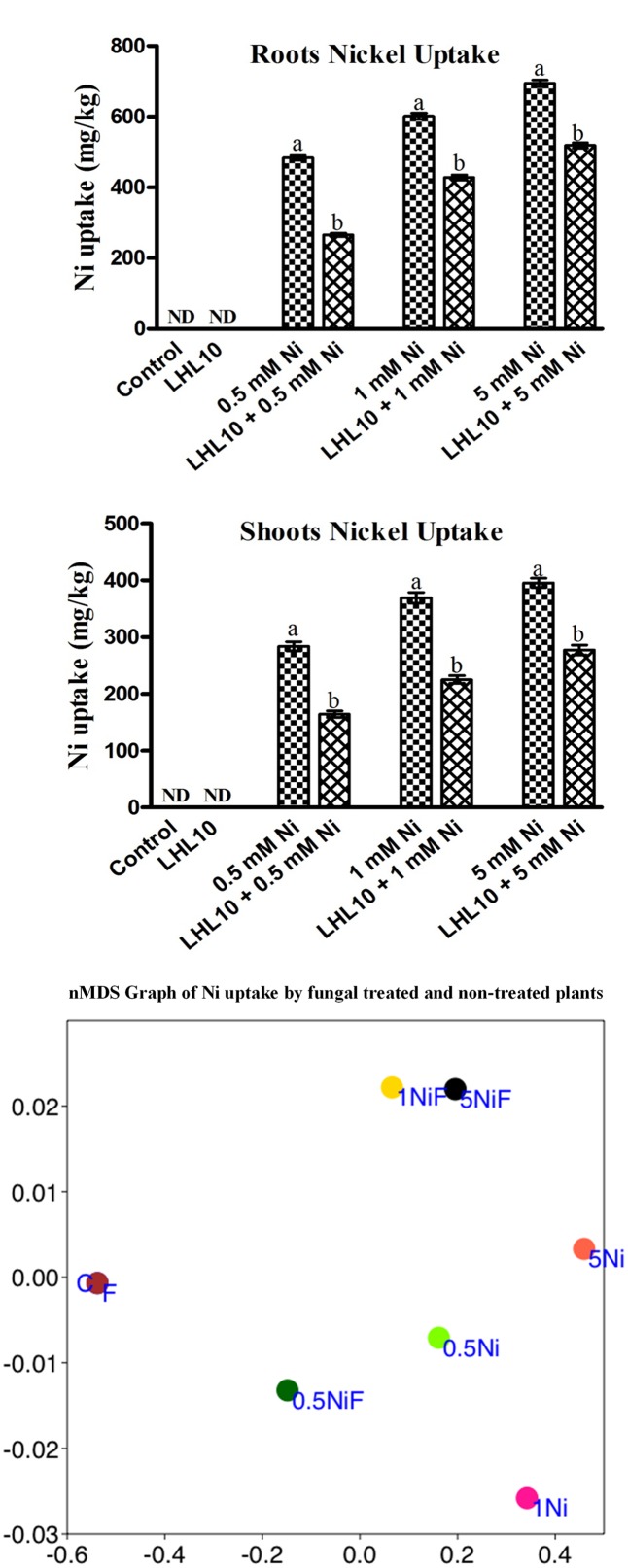
**Ni accumulation in roots and shoots of soybean plants inoculated with and without the fungal endophyte LHL10.** Each value represents the mean ± standard error. Different letters on the bars represent significant difference between the treatments (Tukey’s HSD, *p* < 0.05). A non-metric multidimensional scaling plot portrays Ni uptake by fungal-inoculated plants and non-inoculated plants (C, control; F, only fungal treated; 0.5, 1.0, 5.0 mM; Ni, Nickel-treated plants; NiF, fungal- and nickel-treated plants).

Determination of metal accumulation is important for delineating the role of plants in remediation. Ni was not found to accumulate in the roots or shoots of endophytic fungal-treated and non-treated plants grown in the absence of metal. Non-inoculated plants grown in the soil containing Ni application, accumulated a substantial amount (*p* < 0.05) of Ni in their roots followed by their shoots (**Figure 3** and **Table 3**). However, soybean plants inoculated with *P. formosus* presented lower Ni uptake as compared to inoculated plants, which was 483.66 to 694.66 mg/kg in roots, followed by 284 to 395 mg/kg in shoots under lower and higher stress, respectively. The results revealed that non-inoculated plants accumulated a 30–42 and 35–35% higher amount of Ni in their shoots and roots, respectively, under varied Ni concentrations. The results indicated that Ni accumulated in the order of root > shoot.

**Table 3:** Two-way ANOVA table of the biochemical analysis performed for inoculated and non-inoculated soybean under different concentration of Ni toxicity.

Analysis	Non-inoculated and LHL 10			Ni stress			Interaction		
			
	MS	*P*-value	variance %	MS	*P*-value	variance %	MS	*P*-value	variance %
ABA shoots	3520	0.0001	8.62	115500	0.0001	84.83	8750	0.0001	6.43
ABA Roots	58130	0.0001	9.28	171900	0.0001	82.34	17310	0.0001	8.29
JA Roots	30110	0.0001	12.62	45150	0.0001	56.78	24030	0.0001	30.23
JA Shoots	32510	0.0001	18.90	38950	0.0001	67.93	7449	0.0001	12.99
Root Ni uptake	120600	0.0001	8.33	427900	0.0001	88.71	14030	0.0001	2.91
Shoot Ni uptake	58130	0.0001	11.37	135700	0.0001	84.56	6284	0.0001	3.92
LPO	6.848	0.0001	24.78	5.516	0.0001	59.87	0.5414	0.0472	5.88
GSH	4.815	0.0001	45.81	1.149	0.0001	32.80	0.6473	0.0001	18.48
POD	7083	0.0001	17.11	9973	0.0001	72.26	1399	0.0001	10.14
PPO	41.11	0.0001	80.41	0.05457	0.8841	0.32	1.934	0.0022	11.32


*Two-way ANOVA was followed by a Bonferroni *post hoc* test with a *p* ≤ 0.05 for Ni stress level, LHL10-inoculated and non-inoculated plants, and their interaction, through GraphPad Prism software (version 6.01, San Diego, CA, United States).*

In order to further validate the Ni-detoxifying role of endophytic fungi, nMDS ordination was conducted on the Ni-uptake data for inoculated and non-inoculated plants (**Figure 3**). Control and only fungal-infected plants were found to have the same cluster in the absence of Ni stress. However, as the level of Ni stress increased, the clusters of fungal-treated and non-treated plants became distinct, as shown in **Figure 3**. At 1 mM Ni toxicity, the fungal-treated and non-treated plants did not form a cluster, showing a significant effect of fungal treatment. Both 1 NiF and 5 NiF exhibited a strong correlation by showing a similar distance from each other as compared to other treatments (**Figure 3**). Translocation factor was determined to evaluate the Ni translocation efficiency of soybean plants from root to shoot. TF extended from 0.528 to 0.622 and showed different orders of effect, regardless of metal application and fungal inoculation in soybean plants (**Table 2**). Similarly, there was a decrease in Ni tolerance capability as metal concentration increased. *P. formosus-*inoculated plants had a significantly high TI%, which was around 11.01, 10.57, and 10.82% higher in their respective metal stress (0.5, 1.0, and 5.0 mM) than those of non-inoculated plants (**Table 2**). These results clearly indicate that the interaction of endophytic *P. formosus* with soybean alleviates stress induced by Ni toxicity.

### Association of *P. formosus* Reduces Lipid Peroxidation and Regulates Fatty Acids Composition

**FIGURE 4:**
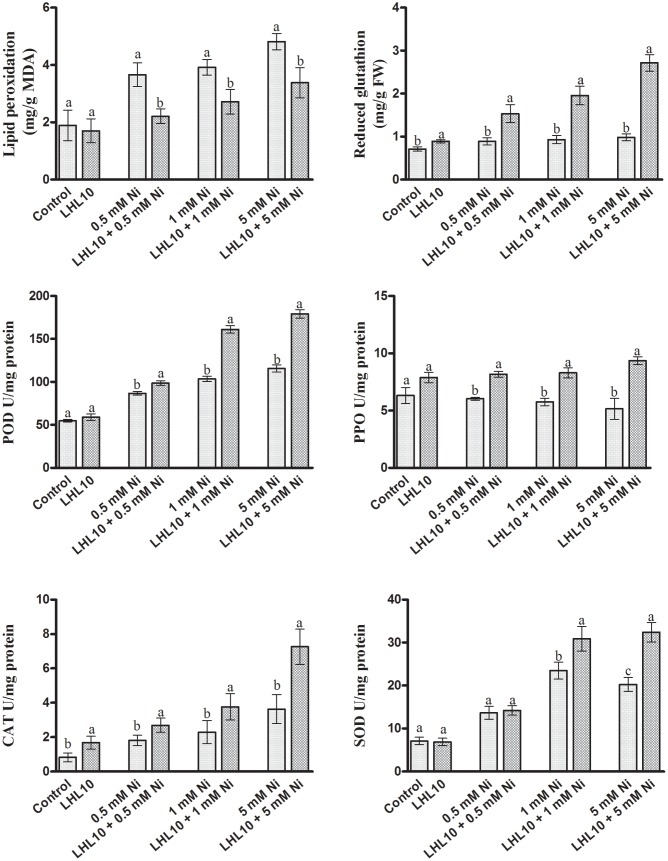
**Effect of LHL10 inoculation on MDA, GSH concentration and POD, PPO, SOD, and CAT activity in soybean shoots under different concentrations of Ni.** Each value represents the mean ± standard error. Different letters on the bars represent significant differences between the treatments (Tukey’s HSD, *p* < 0.05).

Malondialdehyde content, which is an indicator of lipid peroxidation, was evaluated using a spectrophotometer. Normally, metal-induced stress alters the MDA content, which induces lipid peroxidation. Likewise, in the current experiment, the MDA contents in soybean leaves increased as the level of Ni toxicity increased both in endophytic fungal-treated and non-treated plants. However, the MDA content was significantly reduced in plants inoculated with *P. formosus* (*p* < 0.05) under all Ni-stress treatments compared with the non-inoculated plants (**Figure 4**). Under normal conditions, both control and inoculated plants exhibited similar levels of MDA. These results showed that *P. formosus* treatment inhibited the lipid peroxidation process, and consequently, enhanced plant tolerance against Ni toxicity by protecting cell membranes from metal attack. To rectify the reduction in MDA content induced by *P. formosus* association, alteration in essential fatty acids composition under different levels of Ni toxicity was assessed (**Table 4**). The results revealed that Ni toxicity at varying concentrations affects the analyzed fatty acids, including palmitic, stearic, oleic, and linolenic acids. Under the control condition, fungal-inoculated plants had statistically (*p* < 0.05) higher percentages of palmitic acid and stearic acid as compared to non-inoculated plants. Levels of linolenic acid were not statistically significant between *P. formosus-*treated and non-treated plants in the control condition. In contrast, inoculated plants showed a significantly (*p* < 0.05) lower level of palmitic and oleic acid under Ni toxicity as compared to non-inoculated plants. The level of stearic acid was not statistically different between fungal-inoculated and as non-inoculated plants under Ni stress. In the case of linolenic acid, *P. formosus-*infected plants displayed a significantly marked accumulation, with increases of 43, 44, and 66%, respectively, compared with non-treated plants under the respective concentrations of Ni stress.

**Table 4:** Regulation of fatty acids by *Paecilomyces formosus* LHL10 in soybean under Ni- stress.

Treatment	Palmitic	Stearic	Oleic	Linolenic
Control	15.74 ± 0.29^b^	10.76 ± 0.21^b^	40.5 ± 1.91^a^	33.0 ± 2.13^a^
LHL10	17.26 ± 0.35^a^	11.96 ± 0.36^a^	36.02 ± 1.85^b^	34.7 ± 2.536^a^
0.5 mM Ni	20.64 ± 0.58^a^	15.02 ± 1.35^a^	43.41 ± 1.57^a^	20.93 ± 1.83^c^
LHL10 + 0.5 mM Ni	18.67 ± 0.34^b^	13.4 ± 1.72^a^	31.7 ± 1.79^c^	36.23 ± 2.46^a^
1 mM Ni	24.07 ± 0.53^a^	14.9 ± 0.56^a^	43.02 ± 1.58^a^	18.01 ± 2.17^c^
LHL10 + 1 mM Ni	21.34 ± 0.72^b^	13.54 ± 1.67^a^	33.02 ± 1.43^c^	32.1 ± 2.23^a^
5 mM Ni	25.68 ± 0.61^a^	17.95 ± 1.04^a^	47.36 ± 2.05^a^	9.01 ± 1.41^c^
LHL10 + 5 mM Ni	22.87 ± 0.58^b^	15.38 ± 1.32^a^	35.15 ± 2.35^b^	26.6 ± 2.45^a^


*Each value represents the mean ± SD of 11 replicates from three independent experiments. Values in the columns followed by different letters are significantly different at *p* ≤ 0.05 based on the *t-*test.*

### Regulation of Soybean Antioxidant System by *P. formosus* in Response to Ni Exposure

Nickel toxicity in plants triggers oxidative stress through the excessive production of reactive oxygen species (ROS), thus leading to lethal irreversible damage to plant growth and functionality. Therefore, the antioxidant system of soybean plants, including the action of enzymatic antioxidants, was evaluated through spectrophotometry, as they can scavenge ROS efficiently and mitigate their adverse effects. Likewise, in the current experiment, the GSH contents in soybean leaves were found to increase as the Ni toxicity level increased both in endophytic fungal-treated and non-treated plants (**Figure 4**). However, plants inoculated with *P. formosus* had significantly (*p* < 0.05) increased levels of GSH contents under all Ni-stressed treatments compared with non-inoculated plants (**Figure 4** and **Table 3**). Under normal conditions, inoculated plants exhibited slightly increased levels of GSH compared with non-inoculated plants. ROS-induced stress in plant tissues generated by Ni toxicity is mitigated by the synthesis and regulation of antioxidants and related enzymes. Reduced GSH production was significantly boosted in the shoots of endophytic fungal-inoculated plants when exposed to varying concentrations of Ni toxicity, as well as under normal conditions. In terms of the regulation of antioxidant enzymes by endophytic fungal, POD activity was similar in both inoculated and non-inoculated plants in the absence of Ni stress (**Figure 4**). Fungal-infected plant exhibited significantly higher (13–36%) POD activity than non-fungal-treated plants under Ni-induced stress. PPO activity in inoculated plants was increased significantly under Ni-toxicity, with 27 to 44% greater activity than that in non-fungal-treated plants (**Figure 4**). Under the control condition, endophytic fungal-treated plants exhibited 19.9% higher PPO activity than non-inoculated plants. In case of CAT activity, fungal-inoculated plant displayed significant stimulated activity both under control and stress condition. A significant enhancement in CAT activity of inoculated plants was detected at 5.0 mM Ni stress level, which was approximately 50% higher than non-inoculated plants. In terms of SOD activity, inoculated as well as non-inoculated plants showed statistically non-significant activity both under control and 0.5 mM Ni stress. However, significant enhancement (*p* < 0.05) in SOD activity of fungal inoculated plants than non-inoculated plants was observed at 1.0 and 5.0 mM Ni stress levels. The SOD activity in non-inoculated plants went to its maximum at 1 mM Ni stress, but the decreased was observed at highest Ni stress level.

### Regulation of Plant ABA and JA by *P. formosus* under Ni Stress

**FIGURE 5:**
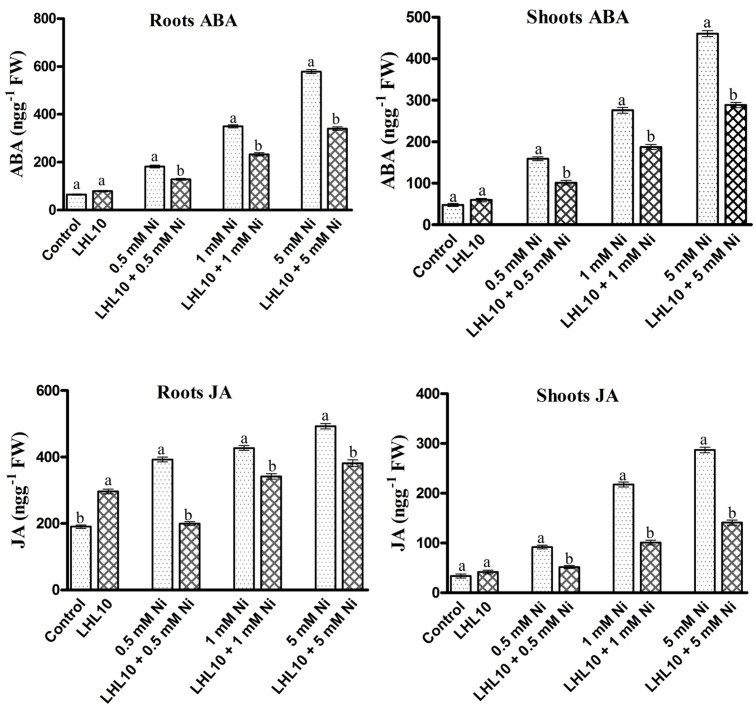
**Regulation of endogenous abscisic acid (ABA) and jasmonic acid (JA) in inoculated and non-inoculated soybean roots and shoots under different levels of Ni stress.** Each value represents the mean ± standard error. Different letters on the bars represent significant differences between treatments (Tukey’s HSD, *p* < 0.05).

Abscisic acid and jasmonic acid are plant phytohormones known for their responsiveness to endogenous stress behavior and enhanced expressed under abiotic stress conditions. In the current experiment, *P. formosus* symbiosis with plants significantly increased the ABA level (*p* < 0.05) in shoots as compared to control plants in the absence of Ni stress (**Figure 5** and **Table 3**). However, upon exposure to Ni stress, endophytic fungal-treated plants exhibited significantly reduced content (32–36%) of ABA compared with non-fungal-infected plants. Likewise, ABA content in the roots was promoted in *P. formosus-*treated plants compared with non-inoculated plants under normal growth conditions. When exposed to Ni stress, soybean roots appeared to be more sensitive and produced higher levels of ABA both in fungal-treated and non-treated plants (**Figure 5**). However, fungal-inoculated plants had significantly reduced levels of ABA in the root during Ni stress compared with non-inoculated plants. These results indicate that interaction with *P. formosus* helped to mitigate the toxic effect of Ni in plants. The JA content in plants followed the same trend as the ABA content. The JA content was significantly (*p* < 0.05) increased in response to Ni stress both in endophyte-treated and non-treated plants. During Ni stress, non-fungal-treated plants exhibited a higher concentration of JA in the roots and shoots, which was increased by 44, 54, and 51%, and 49, 22, and 23%, respectively, compared to fungal-treated plants at concentrations of 0.5, 1.0, and 5.0 mM, Ni, respectively. The down-regulation of endogenous hormones in soybean clearly shows that the interaction with *P. formosus* was beneficial and aided soybean to ameliorate stress induced by heavy metals by triggering their defense system.

## Discussion

In the current study, we assessed the remediation of heavy metals by different endophytic fungal strains (*Penicillium janthinellum* LK5, *P. formosus* LHL10, *Exophiala* sp. LHL08, and *Preussia* sp. BSL10) in Ni-, Cu-, and Cd-contaminated media. The initial investigation suggested that *P. formosus* LHL10 is the most effective strain owing to its higher growth rate in all media contaminated with metal as compared to other strains. *P. formosus* showed higher tolerance to Ni pollution than Cu and Cd. Growth of *P. formosus* in liquid broth indicated that the fungal strain was very effective in Ni removal. The remarkable Ni uptake by *P. formosus* mycelium demonstrates its metal bio-remediating ability which could be attributed either to intercellular absorption or extracellular adsorption (Li et al., 2017). Generally, surface adsorption via ion exchange, extracellular precipitation, hydrolytic adsorption, metal transformation, and sequestration are the favorable biological approaches, adopted by fungi for survival against metal toxicity (Fomina and Gadd, 2014). The remediating process by *P. formosus* is further facilitated with the extracellular production of chemically active substances, in current case phytohormones. Therefore in current study, both inter and extracellular mechanisms seem to be implied simultaneously by *P. formosus* for metal tolerance. Inter-cellular presence of Ni accumulation by *P. formosus* through ICP-MS proves the deposition in vacuole. Whereas, extracellular production of phytohormones (GA and IAA) and existence of biosynthetic genes further re-affirm the metal stress resistance ability. This was also previously validated by the reports of Rajkumar et al. (2012).

The current results showed that *P. formosus* LHL10 showed the existence of GA and IAA biosynthesis related genetic expression. GAs are synthesized from converting mevalonic acid into *geranylgeranyl diphosphate* via *hydroxymethylglutaryl coenzyme A*, *farnesyl diphosphate* and *geranylgeranyl diphosphate* (GGDP). We observed the expression of *GGS2* was detected in RT-PCR, which encodes GGDP synthase principally accountable for providing *GGDP* for GA production. Similarly, *ent-kaurene oxidase (P450-4)* expression indicates that *P. formosus* has cluster of P450 monooxygenase-encoding genes which play crucial role in GAs biosynthesis. *GA20-oxidase* expression was also analyzed in *P. formosus* in the current study and the result showed expression in RT-PCR. *GA20-oxidase* has been reported for in the synthesis of active GAs in *F. fujikuroi* (Tudzynski et al., 2001; Bömke and Tudzynski, 2009). *C13-oxidase (P450-3)* was also analyzed in *P. formosus* and was comparatively less expressed. *C13-oxidase (P450-3)* has the role to catalyze C_13_-hydroxylation of GA_4_ to produce the minor GA1 (Bömke and Tudzynski, 2009). The presence of aforementioned genes involved in the GAs biosynthesis pathway validated the GA producing capability of endophytic *P. formosus* LHL10. Previously, same GA gene cluster has also been reported in *Fusarium fujikuroi*, *Gibberella fujikuroi*, and *Fusarium proliferatum* (Tudzynski et al., 2001; Albermann et al., 2013; Rim et al., 2013). However, there is no information on GA gene biosynthetic pathway in *Paecilomyces* sp. Current report has elucidated it for the first time.

The IAA producing ability of *P. formosus* was rectified via RT-PCR analysis. The IAA biosynthesis related genes expression, i.e., *ALD* and *IAAH* exhibited transcript expressions. The *ALD* genes are reported to be required in tryptophan synthesis, which is the precursor of IAA synthesis as well as involved in stress response (Henke et al., 2016). Similarly the expression of *IAAH* further authenticates the IAA producing ability of endophytic *P. formosus*. As *IAAH* encodes indole-3-acetamide hydrolase that constitute indole-3-acetamide pathway, which leads to the synthesis of IAA through converting tryptophan to indole-3-acetamide, encoded by *IaaM* tryptophan-2-monooxygenase. Indole-3-acetamide is further converted into IAA by the help of *IAM hydrolase* which is encoded by *IaaH* (Tsavkelova et al., 2012). Previously, *Tricholoma vaccinum*, *Fusarium verticillioides*, *Fusarium proliferatum*, and *Saccharomyces cerevisiae* have been reported for possessing IAA biosynthetic pathway (Rao et al., 2010; Tsavkelova et al., 2012; Krause et al., 2015).

The results of this study revealed that the endophytic fungal *P. formosus* has the potential to remediate metal toxicity in metal-polluted environments, and can thus be deployed for mitigating heavy-metal stress in plants in order to improve growth and yield and to clean up the metal-contaminated sites. The production of various biochemical such as GA and IAA by *P. formosus* may contribute to the bioremediation ability of endophytic fungi (Khan et al., 2015a; Saleem et al., 2015). Fungal endophytes that produce IAA have been repeatedly recognized for their ability to counteract the toxic effects of heavy metals (Visioli et al., 2015). Fungal endophytes with metal-resistant capabilities employ different metabolic pathways as well as physiological and molecular strategies for effective intracellular and extracellular detoxification of heavy metals (Deng et al., 2016).

The interaction between endophytes and plants in metal-contaminated soil allows them to grow well and mitigates the toxic effect of the metal (Babu et al., 2014; Sura-de Jong et al., 2015). Fungal endophytes such as *Aspergillus, Penicillium, Fusarium, Paecilomyces, Cladosporium, Lasiodiplodia, Glomerella*, and *Phomopsis* have been recognized for their growth in metal-contaminated soil and their ability to counteract stress induced by heavy metals, such as Cd, Al, Zn, Pb, and Cu (Deng et al., 2014, 2016; Khan et al., 2016). To date, very little information is available regarding endophytic fungi, particularly *P. formosus*, and their symbiotic role in crop plants, especially soybean, and whether they provide any protection or extend tolerance to soybean under Ni toxicity. In this study, we observed severe toxicity induced by Ni contamination in soybean, which significantly retarded plant growth attributes. Our findings showing effects of Ni on soybean growth parameters were in concordance with previous studies by Sirhindi et al. (2016) in *Glycine max*, Siddiqui et al. (2011) in *T. aestivum*, and Rehman et al. (2016) in maize. Conversely, the roots, shoot dry and fresh weight of soybean were remarkably enhanced following inoculation with *P. formosus*. Improvements in biomass and other growth parameters of *Solanum nigrum* were observed following inoculation with *P. lilacinus* under Cd and Pb toxicity (Gao et al., 2010, 2012). The direct vulnerability of non-inoculated soybean roots to Ni stress resulted in poor root growth, probably due to the inhibition of mitosis, which adversely affects the growth parameters of the whole plant (Sirhindi et al., 2016).

Interaction of the plant with *P. formosus* mitigated Ni toxicity and favored plant growth owing to the secretion of plant growth regulatory metabolites such as IAA, and GA, which help to counteract stress induced by metal toxicity. Previous studies have revealed roles for GA and IAA in the modulation and adaptation of plants under severe heavy metal stress (Bashri and Prasad, 2016; Liu et al., 2016). Furthermore, fungal endophytes possess different metal degradation pathways, as well as chelation or sequestration systems, and therefore increase the tolerance of the host plant to heavy metals and assist their growth in metal-polluted soils (Aly et al., 2011; Card et al., 2015). Increases in Ni concentration markedly affected the pigment system of soybean in the current study and resulted in reduced chlorophyll contents. Inhibition of chlorophyll contents by Ni toxicity in tomato and cotton has been reported (Khaliq et al., 2016; Mosa et al., 2016). Decrease in the chlorophyll content can be attributed to the inhibition of proto chlorophyllide reductase and ALA dehydratase enzymes following Ni contamination, which play crucial roles in chlorophyll biosynthesis (Noriega et al., 2007; Gill et al., 2015). In our study, *P. formosus-*inoculated plants exhibited significant increases in chlorophyll content as compared to non-inoculated plants under different concentrations of Ni. Enhanced growth attributes and chlorophyll content in inoculated plants subjected to Ni stress may be due to the reduced levels of MDA and H_2_O_2_ following inoculation with *P. formosus*. Our results are in accordance with those of previous studies (Babu et al., 2014; Padash et al., 2016), which found increased chlorophyll contents in maize and lettuce under Pb and Zn stress following inoculation with endophytic *Trichoderma virens* and *Piriformospora indica* strains, respectively.

To further assess the beneficial effect of *P. formosus* inoculation, it is important to determine the distribution and accumulation of metal uptake in the roots and shoots when evaluating the survival of plants subjected to metal stress (Gao et al., 2015). Here, we found mitigating effects in inoculated soybean plants in terms of a lower accumulation in shoots followed by roots compared with non-inoculated plants. As roots are the primary site of the plant in contact with the soil, they may accumulate more toxic metals (Czerpak et al., 2006). The increased accumulation of nickel in roots might be due to its compartmentalization in root vacuoles (Sharma et al., 2016). The decreased metal accumulation observed in fungal-treated plants further strengthens the finding that *P. formosus* has a higher capability of Ni sorption or degradation during interaction with soybean plants. Furthermore, inoculation or plants with growth-promoting fungal endophytes that are able to produce IAA, organic acids, and siderophores minimized the phytotoxic effects of heavy metals and enhanced metal uptake (Deng et al., 2016). Thus the lower accumulation of Ni indicates that the interaction between the endophytic fungus *P. formosus* and soybean under varying levels of Ni toxicity alleviates the adverse impact of metal-induced stress.

In addition, the present study showed that excessive Ni toxicity alters the activity of metabolic enzymes and indirectly causes oxidative stress. Lipid membrane integrity and activities related to membrane-associated enzymes are markedly affected by heavy metals (Kamran et al., 2015; Sirhindi et al., 2015). Plants tightly regulate their GSH network, which is considered to have a key role in reducing cellular damages (Jozefczak et al., 2012). Disruption of cellular membranes can be ascribed to the production of superoxide radicals $O_{2}^{•-}$ in response to Ni stress. Abundant MDA is generated as a product of polyunsaturated fatty acids during peroxidation of lipid membranes. We found that Ni stress boosted MDA contents in the control plants, which was indicative of serious damage to lipid membranes in response to elevated Ni stress. As reported by Llamas and Sanz (2008) and Gajewska et al. (2012), increased Ni toxicity induces structural damage in rice and wheat lipid membranes, which severely alter their properties and enhance K^+^ efflux from cells. However, MDA accumulation was successfully minimized in *P. formosus-*treated soybean under Ni stress, providing strong evidence that fungal inoculation has the ability to mitigate oxidative injuries.

Our results are in line with previous findings (Khan and Lee, 2013; Khan et al., 2015b) who found the protective effect of the endophytic fungi *P. funiculosum* and *Penicillium janthinellum* on lipid bilayers breakdown under Cu and Al toxicity in tomato plants, respectively. However, it might be assumed from the results of our study that the secretion of various endogenous phytohormones might directly or indirectly enhance plant tolerance through the low generation of ROS. Ni toxicity significantly altered the fatty acid composition of non-treated plants by enhancing the saturation and breakdown of fatty acids, with the exception of linolenic acid. Polyunsaturated fatty acids are extremely sensitive to peroxidation; therefore, their decreased accumulation indicates a low level of Ni-induced oxidative reactions in cellular membranes. Our results are in accordance with those of Gajewska et al. (2012) and Kim et al. (2014) who reported high accumulations of palmitic acid, stearic acid, and oleic acid, and lower accumulation of linolenic in wheat and rice under Ni and Cd stress. Whilst in *P. formosus* treated plants, high levels of linolenic acid were detected in response to high nickel toxicity. From these findings, we can conclude that inoculation with *P. formosus* may lead to the activation of phospholipases as well as phospholipid-derived molecules, which are thought to be involved in plant defense signaling. Phospholipase activation causes the release of alpha-linolenic acid from membrane lipids, which acts as a precursor of JA, a signaling molecule that enhances plant defense systems (Dar et al., 2015; Hou et al., 2016).

Subsequently, our result showed that Ni stress seriously impairs antioxidant components in soybean, such as POD, PPO, CAT, and SOD as well as the low-molecular weight non-protein antioxidant GSH. However, inoculation with *P. formosus* significantly induces antioxidant activities, including POD, PPO, GSH, CAT, and SOD with increasing levels of Ni toxicity. This implies the beneficial interaction with endophytic fungal enables plants to cope with oxidative stress induced by Ni toxicity. The findings of the present study are consistent with those reported by Degola et al. (2015) and Wang et al. (2016), who found enhanced antioxidant activities in maize and *Nicotiana tabacum* plants inoculated with endophytic fungi under heavy-metal stress. GSH is an important antioxidant owing to its reducing potential, which can respond directly as a free radical scavenger. GSH is also a precursor for the synthesis of metal chelating phytochelatins. Hence, its accumulation during conditions of oxidative stress can help protect plant macromolecules, such as lipids, proteins, and DNA either by acting as an electron donor to scavenge ROS, or as organic free radicals, or by the formation of direct adducts with reactive electrophiles (Asada et al., 1994; Sharma et al., 2012). As a precursor of phytochelatins, the higher production of GSH in fungal-inoculated plants might have resulted in their increased biosynthesis. Higher production of phytochelatins and homophytochelatins are considered crucial for detoxification and homeostasis of heavy metals and metalloid toxicity in soybean plants (Vázquez et al., 2009). Therefore, in our study, the enhanced accumulation of GSH in *P. formosus-*treated plants may signify a protective role of endophytic fungal in nickel-induced stress. Similarly, increasing POD activity was observed with increased Ni concentration in inoculated plants, while non-treated plants showed decreased POD activity under nickel toxicity. As reported, metal toxicity leads to the suppression of POD (Hossain and Komatsu, 2013; Xie et al., 2015; Wang et al., 2016). The high level of POD level in endophyte-treated plants under Ni stress is consistent with the findings of Yang et al. (2015), Jiang et al. (2016), and Mendarte-Alquisira et al. (2016), who observed a remarkable increase in the POD concentration of fungal-inoculated *Robinia pseudoacacia* L., *S. nigrum*, and *Festuca arundinacea* plants, respectively, in response to different heavy-metal treatments. POD is considered vital for the dismutation of H_2_O_2_ to water and molecular oxygen. In the current experiment, increased POD activity suggests that the application of endophytic fungi scavenged ROS and helped soybean plants to reduce oxidation-induced damage. Likewise, PPO is thought to play a vital role in abiotic stresses; consequently, the results reported herein show that endophytic fungal-treated plants possess high PPO activity in under Ni toxicity. PPO chelates metals ions and directly encounters ROS during heavy metal stress and can prevent lipid peroxidation by scavenging the lipid alkoxyl radical (Arora et al., 2000; Sharma et al., 2012). Our results were in line with those reported by Hashem et al. (2016) who observed increased PPO activity in fungal-inoculated plants under cadmium stress.

In current study, the SOD activity was detected to enhance significantly in fungal inoculated plants than those of non-inoculated plants under Ni toxicity. Our findings coincides with the results of Rozpdek et al. (2014) and Yang et al. (2015), who detected remarkable SOD activity in endophytic fungal inoculated *Cichorium intybus* L. and *R. pseudoacacia* L. plants, respectively, under Cd, Zn, and Pb stress. The results of the current study demonstrate that fungal inoculation triggered the SOD activity in soybean plant to cope with the super oxide radicals. The higher SOD activity under Ni stress could possibly be related to the high production of superoxide radicals due to the excess of heavy metals ions as well as the *de novo* synthesis of the enzymatic protein (Garg and Kaur, 2013; Yang et al., 2015). Likewise, CAT activity was also significantly enhanced with the increasing Ni stress level in fungal treated than those of non-treated plants. CAT is considered crucial enzyme for the dismutation of H_2_O_2_ to H_2_O and molecular oxygen in the cells. Thus, increase in CAT production in inoculated plants could possibly be the response to the over production of hydrogen peroxide under Ni stress condition – suggesting fungal inoculation successfully alleviated the Ni induced oxidative stress in soybean plants. A number of studies have demonstrated that symbiotic interaction of fungal with host plant maximizes the antioxidant activities in order to strengthen heavy metal tolerance and alleviate oxidative stresses. For instance, Tan et al. (2015) and Khan et al. (2017a) demonstrated that endophytic *A. alternata* RSF-6L and AM *Glomus versiforme* inoculation enhanced CAT activity of *S. nigrum* and *S. photeinocarpum*, respectively, under severe Cadmium contaminated soil. In the current study, the upsurge in the antioxidant activities of inoculated plants under Ni toxicity suggests that endophytic fungal may regulate and activate the genes, which are responsible for encoding for antioxidant enzymes (Wang et al., 2016).

During heavy metal toxicity, plants accumulate high levels of endogenous phytohormones, such as ABA and JA in order to cope with metal-induced stress (Yan et al., 2015; Carrió-Seguí et al., 2016) However, the enhanced biosynthesis of ABA under severe stress leads to leaf senescence and directly inhibits photosynthesis. Hence, the growth rate of plants is suppressed. Deng et al. (2016) reported that high levels of ABA accumulation under multi-metal stress resulted in the inhibition of maize seed germination. Interestingly, in our experiment, *P. formosus-*treated plants resulted in reduced accumulation of ABA in both roots and shoot as compared to non-treated plants during Ni stress. This suggests that endophytic association with plants ameliorates the effects of metal stress. The level of ABA in the root in inoculated plants was higher than that in the shoot, which might be due the direct exposure of roots to metal stress, as it is transported to shoots via apoplastic route to regulate stomatal behavior (Xu et al., 2010). Previous reports rectify our findings that fungal application lowers the accumulation of ABA in plants under abiotic stresses (Khan et al., 2012b; Aroca et al., 2013; Waqas et al., 2015). Maintaining reduced ABA levels in inoculated plants under Ni stress can be accredited to the potential of *P. formosus* to produce GAs, because a similar trend was exhibited by plants in combination with the GA-secreting endophyte *Penicillium resedanum* under abiotic stresses (Khan et al., 2015c). Reduced ABA has often coupled with increased endogenous GA contents. This could be true as the *P. formosus* inoculated plants had significantly higher biomass and shoot length, suggesting pivotive role of GA activation during stress. A similar conclusion was also drawn by Khan and Lee (2013) and Kang et al. (2014), where the GA producing microbes have resulted in reduced ABA and increased GA during abiotic stress conditions.

While assessing the effect of fungal inoculation under nickel toxicity on endogenous JA, which acts as a key regulator in plant defense systems, we observed a tendency for JA to be down-regulated, as compared to that in non-inoculated plants. The reduced level of stress-related hormones, such as ABA and JA compared to control plants suggest that stress is managed better in the fungal-treated plants. The elevated level of JA might inhibit plant growth, as well as suppress GSH synthesis, which is an effective ROS scavenger during stress (Khan A.L. et al., 2017). We studied the increased production of GSH in fungal-treated plants under varying levels of nickel stress. Thus, enhanced GSH synthesis might have helped plants to limit the devastating effects of metal stress by counteracting ROS, and as a result, stress has been resolved and less JA is biosynthesized for plant defense mechanisms. The present results are consistent with those of Kim et al. (2014) who observed decreased levels of endogenous JA in rice under cadmium stress. The current results suggest that endophytic fungus protects plants under Ni stress. Whereas, the cross talk between endogenous hormonal viz. GA, ABA and JA signaling are extremely punitive (Pacifici et al., 2015), that may vary against different environmental stimuli. However, the exact mechanisms of ABA and JA modulation by endophytic fungi under metal stress require further study.

## Conclusion

The current study reports, for the first time, that the GA-secreting endophyte *P. formosus* is a promising alternative for not only improving plant biomass but also significantly protecting host plant form the adverse effects of metal toxicity. Hence, its ability to produce IAA, as reported previously, in combination with GAs might confer tolerance to Ni phytotoxicity by reducing the levels of Ni toxicity in roots and shoots. In addition, it also has induces remarkable increases in growth attributes. Our results revealed that *P. formosus* inoculation enhanced soybean tolerance to Ni via a mechanism affecting the distribution of Ni in soybean tissue and via the induction of hormonal regulation and antioxidant systems. Thus, the association between phytohormone-inducing fungi and soybean may represent a promising strategy for achieving safer and profitable crop production, and to eliminate toxicity from Ni-contaminated soil. However, plant systems are complex and are responsible for controlling intracellular ion levels, including essential nutrients as well as non-essential metals. Therefore, in future studies, emphasis should be given to the role of *P. formosus* in enhancing macro and micro-nutrients under metal toxicity, because these nutrients have repeatedly been reported to enhance metals tolerance or accumulation potential in plants. Additionally, further molecular studies and investigating of transcriptional level work studies are recommended for an in-depth understanding of *P. formosus* association with host plants in metal-contaminated soil as well as Ni-polluted field trials to assess the role of endophytic fungal on a large scale.

## Author Contributions

SB, AK, RS, S-MK conceived and designed the experiments; SB and SA performed the experiments; AK and I-JL analyzed the data; I-JL contributed reagents/materials/analysis tools; SB, AK, and RS wrote the paper.

## Conflict of Interest Statement

The authors declare that the research was conducted in the absence of any commercial or financial relationships that could be construed as a potential conflict of interest. The reviewer VVY and handling Editor declared their shared affiliation, and the handling Editor states that the process nevertheless met the standards of a fair and objective review.
