# Supplementary material for: Endophytic Paecilomyces formosus LHL10 Augments Glycine max L. Adaptation to Ni-Contamination through Affecting Endogenous Phytohormones and Oxidative Stress
Source: Front Plant Sci. 2017 May 29;8:870. doi: 10.3389/fpls.2017.00870 (PMC5447229; doi:10.3389/fpls.2017.00870)
Supplement: Supplementary file 1 [file Table_1.docx]

**Table S1:** Primers used for expression of various transcripts related to phytohormonal production of *Paecilomyces formosus* LHL10 using real time RT-PCR

| Function | Primer | Gene Name | Sequence |
| --- | --- | --- | --- |
| Indole acetic acid (IAA) | ALD | *aldehyde dehydrogenase* | F- GAA AGC TCT TGG AGC AGG TG  R- TGG ACT GTA GCA CCC TCC TT |
|  | IAAH | *Indole-3-acetamide hydrolase* | F- TTA CTG TTC TCT GGA CCC ACA ACC  R- ATC AGG TTG AAC AGC ACA AAG TCGC |
| Gibberellins  (GA) | P450-4 | *ent-desaturase oxidase* | F- CCAAACTCCTCGGACATCACTTTG  R- GTGTCGTAGGTCGAACCCATAGC |
|  | P450-3 | *C13- oxidase* | F- TGCTCACAAGACTGCAAACC  R- CAGGTAAGTGCCGAAGAAGC |
|  | GA 20-oxidase | *GA 20-oxidase* | F- ATGTGG(CT)(AC)NGA(AG)- GGNTT(CT)AC  R- GT(AG)TGNGCNGCNAGNCCCAT |
|  | GGS2 | *Geranylgeranyl-diphosphate synthase* | F- AAGTGGGCTGTGGGGATCGAA  R- CTCTTATCTGGAAATACTGCC |
